# Long-term (2–5 years) adverse clinical outcomes associated with ZES versus SES, PES and EES: A Meta-Analysis

**DOI:** 10.1038/s41598-017-06705-y

**Published:** 2017-07-25

**Authors:** Pravesh Kumar Bundhun, Akash Bhurtu, Manish Pursun, Mohammad Zafooruddin Sani Soogund, Abhishek Rishikesh Teeluck, Wei-Qiang Huang

**Affiliations:** 1 Institute of Cardiovascular Diseases, the First Affiliated Hospital of Guangxi Medical University, Nanning, Guangxi 530027 P. R. China; 2 Guangxi Medical University, Nanning, Guangxi 530021 P. R. China

## Abstract

Several previously published trials comparing Zotarolimus Eluting Stents (ZES) with Sirolimus Eluting Stents (SES), Paclitaxel Eluting Stents (PES) or Everolimus Eluting Stents (EES) at a follow up period of 1 year, were continually being followed up in order to assess the long-term outcomes. In this meta-analysis, we aimed to compare the long-term (2–5 years) adverse clinical outcomes which were associated with ZES versus SES, PES and EES following Percutaneous Coronary Intervention (PCI). Risk Ratios (RR) with 95% Confidence Intervals (CIs) were generated and the analysis was carried out by the RevMan 5.3 software. In this analysis with a total number of 17,606 participants, ZES and EES were associated with similar adverse outcomes including Stent Thrombosis (ST), myocardial infarction (MI), major adverse cardiac events and repeated revascularization. When ZES were compared with SES and PES during the long-term, MI and definite or probable ST were significantly lower with ZES, with RR: 1.35, 95% CI: 1.17–1.56; P = 0.0001 and RR: 1.91, 95% CI: 1.33–2.75; P = 0.0004 respectively whereas the other adverse outcomes were similarly manifested. Future research should be able to confirm this hypothesis.

## Introduction

Coronary artery disease (CAD) affects a large number of people annually. Coronary stents are special devices that are placed within narrow coronary arteries to keep them open so that the heart is supplied with a sufficient amount of blood. This practice may reduce symptoms and prevent heart attacks.

Since recent studies have shown an early hospital discharge to be safe following Percutaneous Coronary Intervention (PCI)^1^, revascularization with the implantation of Drug Eluting Stents (DES) has become a common option in the general population with CAD.

DES mainly consist of three parts: the platform of the stent (mesh-like design), a polymer coating to bind the drug to the stent, and the drug itself. The drug blocks cell proliferation within coronary arteries and therefore inhibits neointimal growth thereby preventing restenosis^2–5^.

Different types of DES such as Sirolimus Eluting Stents (SES) [manufacturer: Cordis, platform: BX Velocity, polymer: persistent, drug: sirolimus, mechanism of action: cytostatic], Paclitaxel Eluting Stents (PES) [manufacturer: Boston Scientific, platform: Express and Liberte, polymer: persistent, drug: paclitaxel, mechanism of action: cytostatic], Everolimus Eluting Stents (EES) [manufacturer: Abbott, platform: Multi-Link Vision, polymer: persistent, drug: everolimus, mechanism of action: cytostatic] and Zotarolimus Eluting Stents (ZES) [manufacturer: Medtronic, platform: Driver, polymer: persistent, drug: zotarolimus, mechanism of action: cytostatic]^6^ are available.

Several trials have compared Stent Thrombosis (ST) and other adverse clinical outcomes which were associated with different types of individual DES^7^. SES were compared with PES^8^ and EES were compared to non-everolimus eluting DES^9^. However, controversies have been observed when ZES were compared with SES, PES and EES during the short and long term follow up periods respectively.

A previously published meta-analysis comparing ZES with SES or PES showed the former not to be superior to PES, but were inferior to SES in terms of angiographic outcomes and repeated revascularization^10^. Another meta-analysis comparing ZES with SES showed the latter to be superior to ZES in terms of Target Lesion Revascularization (TLR) and Major Adverse Cardiac Events (MACEs) without significantly affecting Target Vessel Revascularization (TVR), ST, cardiac death or Myocardial Infarction (MI)^11^. Nevertheless, these meta-analyses were limited to a shorter follow up period of less than 2 years. Newer research with a longer follow up period (≥2 years) were required to assess these outcomes.

However, when newer trials with longer follow up periods were published, the superiority of SES was lost in comparison to ZES. Recently, the Patient Related OuTcomes with Endeavor versus Cypher Stenting Trial (PROTECT) trial showed ZES to significantly reduce ST and composite endpoint of death or MI at 4 years follow up^12^.

Several of the previously published trials which assessed outcomes at 1 year follow-up were continually being studied during the long-term (≥2 years). In addition, other newer trials with longer follow up periods were recently published. Therefore, in this analysis, we aimed to compare the long-term (2–5 years) adverse clinical outcomes which were associated with ZES versus SES/PES and EES following PCI.

## Methods

### Data Sources and Search Strategy

Medical Literature Analysis and Retrieval System Online (MEDLINE) database, the Cochrane database and the EMBASE (www.sciencedirect.com) database were the main electronic databases which were searched for trials (published in English) comparing ZES with SES or PES or EES using the following terms: ‘zotarolimus eluting stents and X’ whereby X was interchangeable with ‘sirolimus eluting stents, paclitaxel eluting stents and everolimus eluting stents’. The term ‘percutaneous coronary intervention’ and the abbreviations ‘ZES, SES, PES, EES and PCI’ were also alternatively used in this search strategy. Related reference lists were also reviewed for relevant trials.

### Inclusion and Exclusion Criteria

Randomized Controlled Trials (RCTs) were considered relevant for this analysis if they compared ZES with SES, PES or EES, and if they reported ST and/or other adverse outcomes as their endpoints during a follow up period of 2 or more years (≥2 years).

Studies were excluded if: they were non-RCTs (meta-analyses, observational cohorts, case-control studies and letters of correspondence), they did not compare ZES with either SES, PES or EES, they did not report ST or other adverse clinical outcomes as their endpoints, outcomes were followed up for period of less than 2 years, and they were studies that involved the same trial or they were duplicated studies.

### Outcomes, Definitions and Follow ups

The primary outcome was ST defined by the Academic Research Consortium (ARC)^13^ and included total ST (definite and probable), as well as definite and probable ST separately.

Definite ST had the following features: ST was confirmed by angiography, the thrombus was formed in the coronary stent or it was 5 mm around the stent along with the following: acute ischemia at rest, electrocardiogram showing new onset of ischemia, typical increase and decrease in cardiac markers, occlusive and non-occlusive thrombus, and evidence of recent thrombus formation at autopsy or thrombectomy.

Probable ST had the following features: Intracoronary ST which was possible because unexplained death occurred within the first 30 days, intracoronary ST which was possible due to any MI which was responsible for acute ischemia without any angiographic confirmation.

Secondary outcomes included: All-cause mortality (cardiac and non-cardiac causes), cardiac mortality (death due to cardiac causes), MI, MACEs (death, MI and revascularization), TVR and TLR.

In this analysis, the outcomes were assessed during a long-term follow up period (2–5 years) as shown in Table 1.

**Table 1:** Reported outcomes and follow up periods.

Trials	Reported outcomes	Follow up period
ENDEAVOR III^16^	Death, cardiac death, MI, definite/probable ST, TLR, TVR, MACEs	5 years
ENDEAVOR IV^17^	Death, cardiac death, MI, TLR, TVR, MACEs, definite/probable ST	5 years
ISAR TEST 2^18^	Death, MI, MACEs, definite/probable ST	2 years
PRODIGY^19^	Death, MI, TVR, definite/probable ST, MACEs	2 years
PROTECT^12^	Death, cardiac death, MI, TLR, TVR, MACEs, definite/probable ST	4 years
SORT OUT III^20^	Death, cardiac death, MI, MACEs, TVR, TLR, definite ST	5 years
ZEST^21^	Death, cardiac death, MI, MACEs, TLR, TVR, definite or probable ST	2 years
PRISON III^22^	Death, cardiac death, MI, TVR, MACEs, definite or probable ST	3 years
ZOMAxx I^23^	Death, cardiac death, MI, TVR, TLR, MACEs, definite or probable ST	5 years
RESOLUTE^24^	Death, cardiac death, MI, TVR, TLR, MACEs, definite or probable ST	4 years
TWENTE^25^	Death, cardiac death, MI, TVR, TLR, MACEs, definite or probable ST	2 years
TWENTE II^26^	Death, cardiac death, MI, TVR, TLR, MACEs, definite or probable ST	2 years

Abbreviations: ST: stent thrombosis, TLR: target lesion revascularization, TVR: target vessel revascularization, MACEs: major adverse cardiac events, MI: myocardial infarction.

### Data Extraction and Quality Assessment

Five authors (PKB, AB, MP, MZSS and ART) carefully assessed the trials which were considered eligible for this analysis. Their methodological qualities were also assessed in accordance to the seven criteria which were linked to the Cochrane Collaboration^14^. A score of 2 points was allotted for a low risk of bias, whereas a score of 0 was allotted for a high risk. Unclear bias was allotted a score of 1. A total score of 14 points (7 criteria × 2 points) implied a very low bias risk. Grades were also given with reference to the scores which were obtained: Grade A (score: 11–14), grade B (score: 8–10), grade C (score: 5–7), grade D (score: 4–6), and grade E (score: 0–3) as shown in Table 2.

**Table 2:** Bias risk assessment of the trials with reference to the Cochrane Collaboration.

Trials	A	B	C	D	E	F	G	Total score	Bias grade
ENDEAVOR III	2	1	2	1	2	1	1	10	B
ENDEAVOR IV	2	1	1	1	2	1	1	9	B
ISAR TEST 2	2	2	2	1	2	1	1	11	A
PRODIGY	2	1	1	1	2	1	1	9	B
PROTECT	2	2	2	2	2	1	1	12	A
SORT OUT III	2	2	1	2	2	1	1	11	A
ZEST	2	2	1	1	2	1	1	10	B
PRISON III	2	2	2	1	2	1	1	11	A
ZOMAxx I	2	2	2	2	2	1	1	12	A
RESOLUTE	2	2	2	1	2	1	1	11	A
TWENTE	2	1	1	1	2	1	1	9	B
TWENTE II	2	2	2	1	2	1	1	11	A

The seven components recommended by the Cochrane Collaborations to assess bias risk: A: Sequence generation B: Allocation sequence concealment C: Blinding of participants and personnel D: Blinding of outcome assessment E: Incomplete outcome data F: Selective outcome reporting G: Other potential sources of bias.

Information and data concerning periods of the patients’ enrollment, the total number of patients who were treated with ZES versus SES, PES or EES respectively, the clinical outcomes (ST and other adverse outcomes) which were reported, the total length of follow up periods, participants’ baseline features, and the total number of events which occurred with each outcome respectively, were systematically extracted. The sixth author’s (WQH) role was to resolve any disagreement which followed during this data extraction process.

### Statistical Analysis

The PRISMA study guideline was used since this present study is a meta-analysis of randomized trials^15^. In this meta-analysis, heterogeneity across the trials were assessed by:

- The Cochrane Q-statistic test (P ≤ 0.05 is statistically significant);
- The I^2^-statistic test (a low heterogeneity was indicated by a low percentage of I^2^, whereas a higher heterogeneity was represented by higher values of I^2^).

In addition, a fixed effects model (if I^2^ < 50%) or a random effects model (if I^2^ > 50%) was used during the statistical analysis based on the I^2^ value which was obtained.

Risk Ratios (RR) with 95% Confidence Intervals (CIs) were used as the statistical parameters and the subgroup analyses were carried out by the latest RevMan 5.3 software.

The number needed to treat (NNTs) for the investigated events were also calculated using the formula: NNT = 1/ARR whereby ARR represented the absolute risk reduction. ARR was calculated by subtracting the experimental event rate from the control event rate. ARR = (Control event rate) – (Experimental event rate).

The optimum information size (OIS) was also calculated for each analysis. OIS was defined as the minimum amount of information which was required to reach reliable conclusions in a meta-analysis. Estimating the OIS might help to determine whether there was sufficient data to draw reliable conclusions. OIS was determined by the Trial Sequential Analysis (TSA) software which is freely available at www.ctu.dk/tsa.

Publication bias (which was assessed based on the shape and symmetry of the funnels) was estimated using funnel plots which were derived from the RevMan software.

Board review for ethical approval was not required for this analysis.

## Results

### Search Outcomes

**Figure 1:**
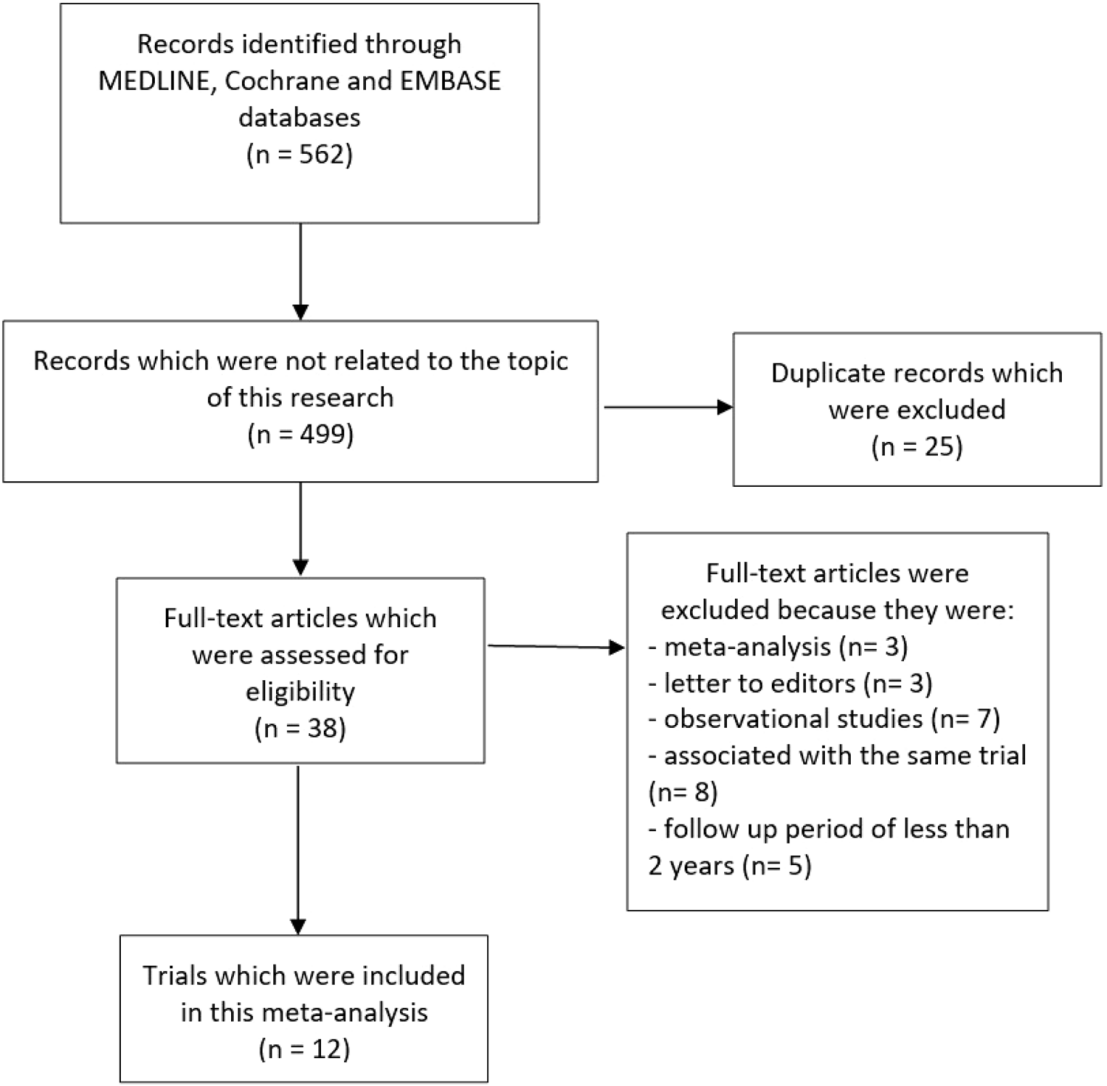
Flow diagram representing the study selection.

Five hundred and sixty-two (562) articles were obtained from the above-mentioned electronic databases. After carefully reviewing the summaries (abstracts) and titles, 499 articles were spontaneously eliminated since they were not associated to this current idea. Another 25 articles were further removed since they were duplicated studies. Thirty-eight (38) full text articles were assessed for eligibility. The full-text articles were again carefully reviewed, whereby a further 26 articles were eliminated since: three articles were meta-analyses and letters of correspondence respectively, 7 articles were observational studies, 8 research articles were associated with the same trial whereas 5 articles had a follow up period of less than 2 years. Finally, only 12 trials^12, 16–26^ were included in this current analysis (Fig. 1).

### General features of the trials which were included in this analysis

A total number of 17,606 patients (8176 patients who were treated by ZES and 9430 patients who were treated by SES, PES or EES) were included in this analysis. The patients’ enrollment time-periods, and the number of patients which were extracted from each trial have been summarized in Table 3.

**Table 3:** General features of the trials which were included in this analysis.

Trials	Patients’ enrollment period	Type of DES in study group	Total no of patients treated with ZES (n)	Total no of patients treated with SES/PES or EES (n)
ENDEAVOR III	2004–2010	SES	307	108
ENDEAVOR IV	2005–2006	PES	722	718
ISAR TEST 2	2006–2008	SES	339	335
PRODIGY	2006–2008	PES, EES	500	500, 501
PROTECT	2007–2008	SES	1174	1236
SORT OUT III	2006–2009	SES	1162	1170
ZEST	2006–2008	SES, PES	883	1762
PRISON III	2007–2010	SES	150	154
ZOMAxx I	2004–2005	PES	199	197
RESOLUTE	2008	EES	1140	1152
TWENTE	2008–2012	EES	695	692
TWENTE II	2010–2012	EES	905	905
Total no of patients (n)			8176	9430

Abbreviations: DES: drug eluting stents, ZES: zotarolimus eluting stents, SES: sirolimus eluting stents, PES: paclitaxel eluting stents, EES: everolimus eluting stents.

PROTECT trial consisted of the largest number of patients in comparison to the other trials. However, since all of the other trials which were included had a smaller number of patients, an adjustment of the number of patients which were extracted from the PROTECT trial was required in order not to influence or affect the result of this analysis. Therefore, only patients with diabetes mellitus were extracted from the PROTECT trial and included in this analysis.

### Baseline features of the participants

The baseline features of the participants were summarized (Table 4).

**Table 4:** Baseline features of the patients.

Trials	Mean age	Males (%)	Ht (%)	Ds (%)	Cs (%)	DM (%)
	Z/D	Z/D	Z/D	Z/D	Z/D	Z/D
ENDEAVOR III	61.4/61.7	65.3/81.4	70.7/74.3	83.5/86.7	66.5/75.2	29.7/28.3
ENDEAVOR IV	63.5/63.6	66.9/68.5	79.4/82.6	81.4/84.8	62.6/60.4	31.2/30.5
ISAR TEST 2	67.2/66.6	75.5/77.3	67.6/63.9	65.5/69.0	18.0/17.3	26.3/27.2
PRODIGY	68.0/68.0	78.0/78.0	69.0/73.0	53.0/59.0	26.0/22.0	24.0/28.0
PROTECT	62.3/62.1	77.0/76.0	65.0/63.0	62.0/63.0	25.0/25.0	100/100
SORT OUT III	64.3/64.3	73.0/74.0	54.0/51.0	70.0/68.0	32.0/32.0	15.0/14.0
ZEST	62.1/62.2	64.2/65.2	65.9/63.4	51.9/50.0	25.4/27.7	30.4/27.9
PRISON III	—	—	—	—	—	—
ZOMAxx I	63.0/63.0	75.0/77.0	69.0/67.0	78.0/72.0	24.0/19.0	22.0/26.0
RESOLUTE	64.4/64.2	76.6/77.2	71.1/71.3	64.0/67.7	26.5/26.5	23.5/23.4
TWENTE	63.9/64.5	72.5/72.6	55.4/55.8	57.0/61.4	25.3/23.6	22.7/20.6
TWENTE II	63.9/63.9	73.4/72.6	55.2/53.5	46.1/47.5	23.6/25.5	18.4/17.3

Abbreviations: Z: zotarolimus eluting stents, D: sirolimus/paclitaxel or everolimus eluting stents, Ht: hypertension, Ds: dyslipidemia, Cs: current smoker, DM: diabetes mellitus.

The participants had a mean age ranging from 61.4 years to 68.0 years. Most of the patients were males with a percentage reaching up to 81.4% in one trial and above 60% in all the trials. According to Table 4, there were no significant differences in the baseline features among patients who were treated by ZES versus SES, PES or EES respectively.

### Long-term Stent Thrombosis which were observed with ZES versus SES or PES

**Figure 2:**
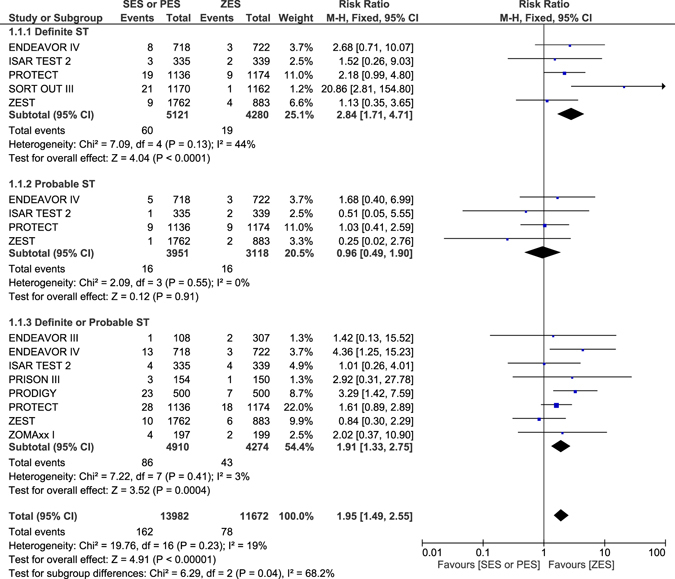
Long term Stent Thrombosis which was associated with ZES versus SES or PES.

Results of this analysis showed ZES to be associated with a significantly lower definite or probable ST with RR: 1.91, 95% CI: 1.33–2.75; P = 0.0004 during this long-term follow up period. Definite ST was also significantly lower in patients who were treated by ZES with RR: 2.84, 95% CI: 1.71–4.71; P < 0.0001 whereas a similar rate of probable ST was observed between ZES and SES or PES with RR: 0.96, 95% CI: 0.49–1.90; P = 0.91 as shown in Fig. 2.

### Long-term Secondary Clinical Outcomes which were observed with ZES versus SES or PES

**Figure 3:**
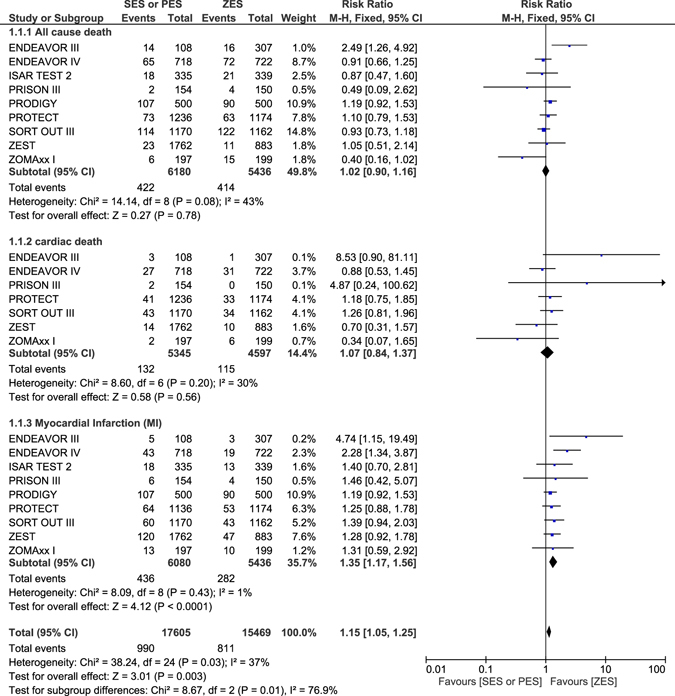
Long term Mortality and Myocardial Infarction which were associated with ZES versus SES or PES.

Other clinical outcomes were also analyzed. Similar all-cause death and cardiac death were observed with ZES and SES or PES during this long-term follow up period with RR: 1.02, 95% CI: 0.90–1.16; P = 0.78 and RR: 1.07, 95% CI: 0.84–1.37; P = 0.56 respectively. Nevertheless, ZES were associated with a significantly lower risk of MI with RR: 1.35, 95% CI: 1.17–1.56; P < 0.0001 (Fig. 3).

**Figure 4:**
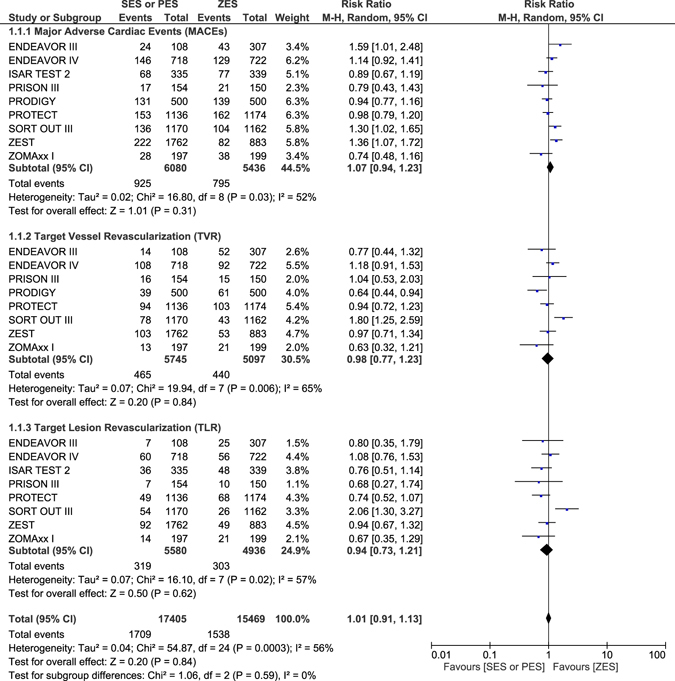
Long term Major adverse events and repeated revascularization which were associated with ZES versus SES or PES.

MACEs, TVR and TLR were similarly manifested with RR: 1.07, 95% CI: 0.94–1.23; P = 0.31, RR: 0.98, 95% CI: 0.77–1.23; P = 0.84 and RR: 0.94, 95% CI: 0.73–1.21; P = 0.62 respectively as shown in Fig. 4. Results of this analysis have been tabulated (Table 5).

**Table 5:** Results of this analysis.

Outcomes analyzed	NNT	OIS	RR with 95% CI	P value	I^2^ (%)
All-cause mortality	151	24,785	1.02 [0.90–1.16]	0.78	43
Cardiac death	151	Infinity	1.07 [0.84–1.37]	0.56	30
Myocardial Infarction	50	2547	1.35 [1.17–1.56]	0.0001	1
Definite or probable ST	140	4554	1.91 [1.33–2.75]	0.0004	3
Probable ST	909	61,489	0.96 [0.49–1.90]	0.91	0
Definite ST	137	2383	2.84 [1.71–4.71]	0.0001	44
MACEs	111	25,149	1.07 [0.94–1.23]	0.31	52
TVR	233	47,528	0.98 [0.77–1.23]	0.84	65
TLR	435	162,522	0.94 [0.73–1.21]	0.62	57

Abbreviations: NNT: number needed to treat, OIS: optimal information size, ST: stent thrombosis, RR: risk ratios, CI: confidence intervals, MACEs: major adverse cardiac events, TVR: target vessel revascularization, TLR: target lesion revascularization.

### Long-term adverse clinical outcomes which were observed with ZES versus SES alone

Another analysis was carried out but this time SES and PES were separately analyzed.

**Figure 5:**
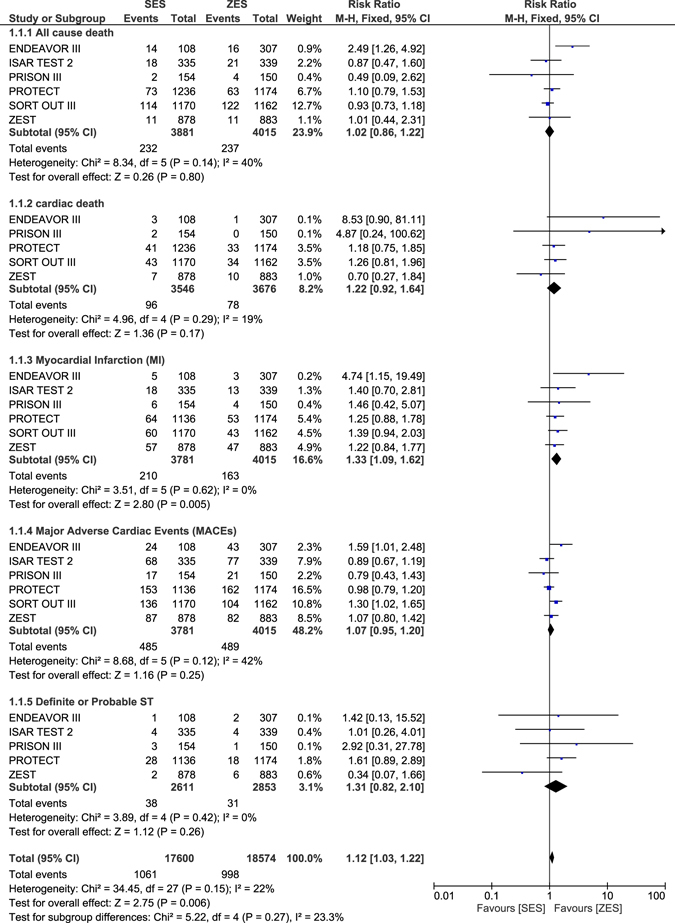
Long-term adverse clinical outcomes which were associated with ZES versus SES alone (part 1).

**Figure 6:**
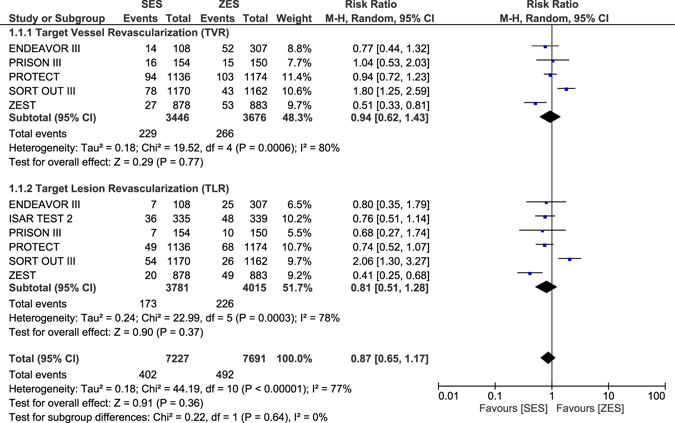
Long-term adverse clinical outcomes which were associated with ZES versus SES alone (part 2).

When ZES were compared with SES alone, all-cause death, cardiac death, MACEs, and definite or probable ST were not significantly different with RR: 1.02, 95% CI: 0.86–1.22; P = 0.80, RR: 1.22, 95% CI: 0.92–1.64; P = 0.17, RR: 1.07, 95% CI: 0.95–1.20; P = 0.25 and RR: 1.31, 95% CI: 0.82–2.10; P = 0.26 respectively as shown in Fig. 5. TVR and TLR were also not significantly different with RR: 0.94, 95% CI: 0.62–1.43; P = 0.77 and RR: 0.81, 95% CI: 0.51–1.28; P = 0.37 respectively as shown in Fig. 6. However, MI significantly favored ZES with RR: 1.33, 95% CI: 1.09–1.62; P = 0.005 (Fig. 5).

### Long-term adverse clinical outcomes which were observed with ZES versus PES alone

**Figure 7:**
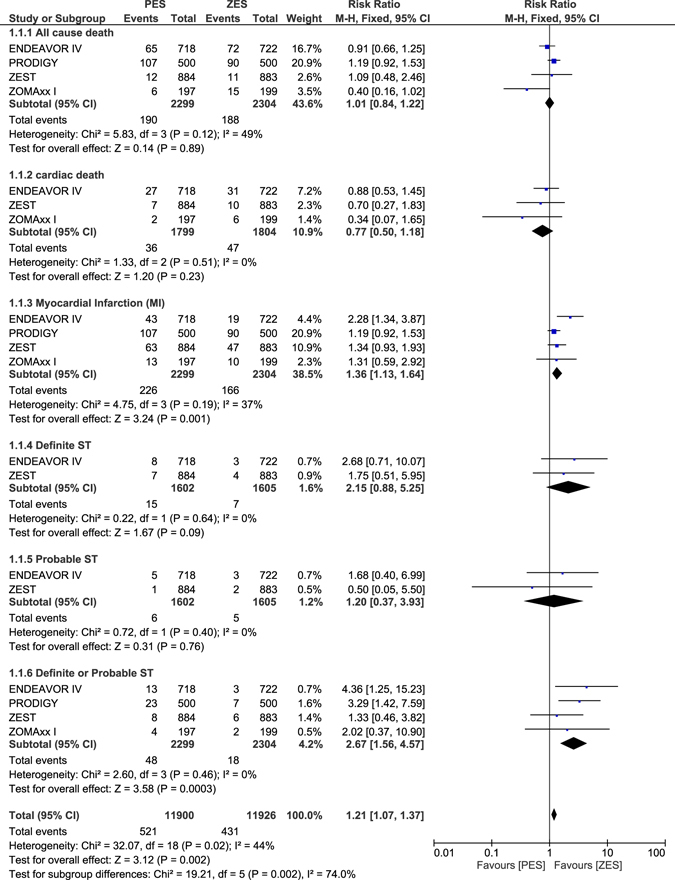
Long-term adverse clinical outcomes which were associated with ZES versus PES alone (part 1).

When ZES was compared to PES alone, all-cause mortality and cardiac death were not significantly different with RR: 1.01, 95% CI: 0.84–1.22; P = 0.89 and RR: 0.77, 95% CI: 0.50–1.18; P = 0.23 respectively. However, MI and definite or probable ST significantly favored ZES with RR: 1.36, 95% CI: 1.13–1.64; P = 0.001 and RR: 2.67, 95% CI: 1.56–4.57; P = 0.0003 respectively as shown in Fig. 7.

**Figure 8:**
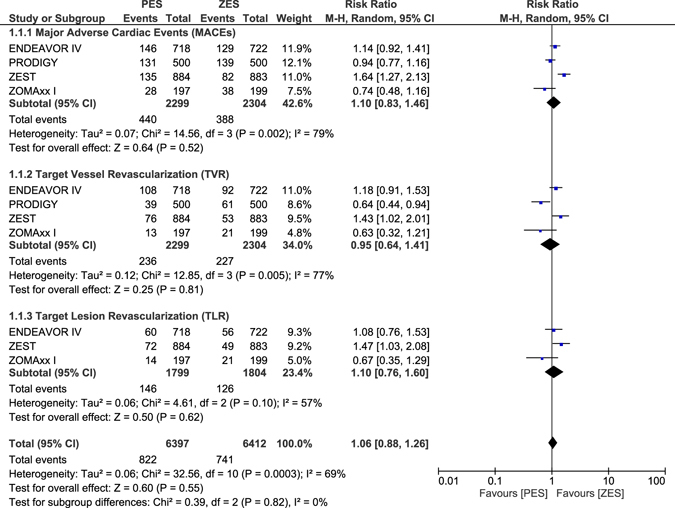
Long-term adverse clinical outcomes which were associated with ZES versus PES alone (part 2).

MACEs, TVR and TLR were not significantly different between ZES and PES with RR: 1.10, 95% CI: 0.83–1.46; P = 0.52, RR: 0.95, 95% CI: 0.64–1.41; P = 0.81 and RR: 1.10, 95% CI: 0.76–1.60; P = 0.62 respectively as shown in Fig. 8.

Table 5 also listed the NNT values and the OIS values which were associated with each outcome respectively. The primary outcomes in this analysis were definite and probable ST whereas the secondary outcomes were the other adverse clinical outcomes. According to the values of OIS obtained, the sample size was sufficient to draw out conclusions for definite stent thrombosis, definite or probable stent thrombosis and most of the other adverse outcomes.

### Adverse outcomes which were observed with ZES versus SES or PES at 2 years follow-up

**Figure 9:**
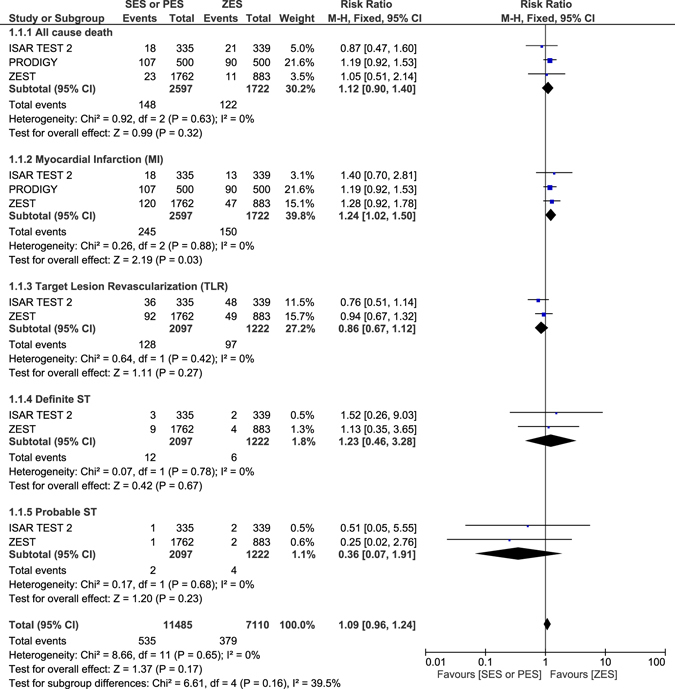
Adverse clinical outcomes which were associated with ZES versus SES or PES at 2-year follow-up (part 1).

**Figure 10:**
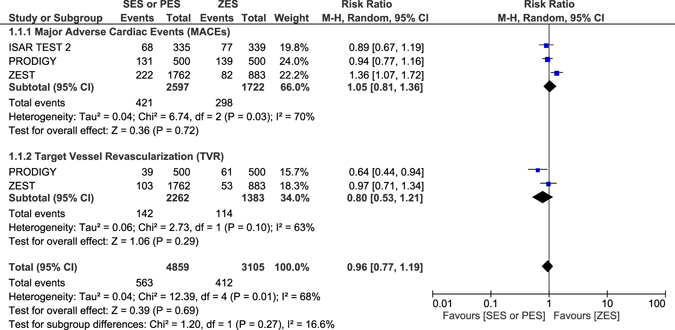
Adverse clinical outcomes which were associated with ZES versus SES or PES at 2-year follow-up (part 2).

Another separate analysis was carried out with respect to the follow-up periods. All the trials which had the same follow-up periods were compared together. At 2 years follow-up period, all-cause mortality, TLR, definite and probable ST were not significantly different between ZES versus SES or PES with RR: 1.12, 95% CI: 0.90–1.40; P = 0.32, RR: 0.86, 95% CI: 0.67–1.12; P = 0.27, RR: 1.23, 95% CI: 0.46–3.28; P = 0.67 and RR: 0.36, 95% CI: 0.07–1.91; P = 0.23 respectively as shown in Fig. 9. MACEs and TVR were also not significantly different with RR: 1.05, 95% CI: 0.81–1.36; P = 0.72 and RR: 0.80, 95% CI: 0.53–1.21; P = 0.29 respectively (Fig. 10). MI significantly favored ZES with RR: 1.24, 95% CI: 1.02–1.50; P = 0.03 (Fig. 9).

### Adverse outcomes which were observed with ZES versus SES or PES at 4–5 years follow-up

**Figure 11:**
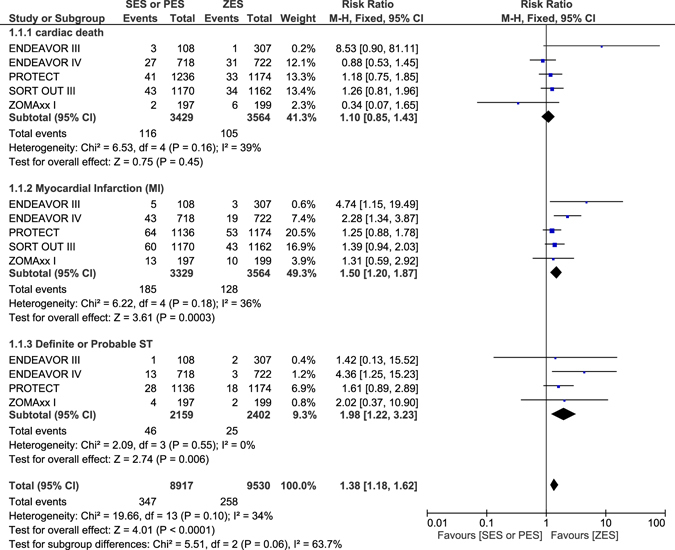
Adverse clinical outcomes which were associated with ZES versus SES or PES at 4–5 years follow-up (part 1).

**Figure 12:**
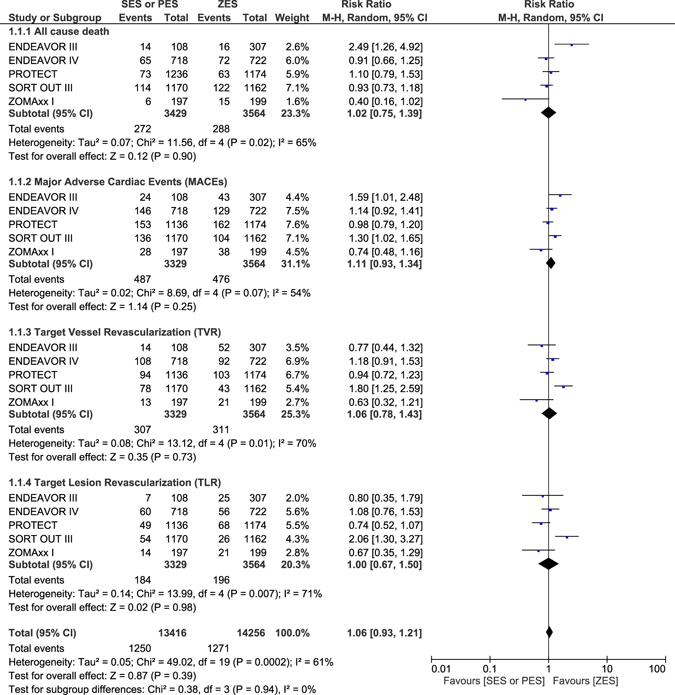
Adverse clinical outcomes which were associated with ZES versus SES or PES at 4–5 years follow-up (part 2).

During a follow up period of 4 to 5 years, cardiac death was not significantly different between ZES and SES/PES with RR: 1.10, 95% CI: 0.85–1.43; P = 0.45. MI significantly favored ZES with RR: 1.50, 95% CI: 1.20–1.87; P = 0.0003. Definite or probable ST were also significantly lower with ZES, with RR: 1.98, 95% CI: 1.22–3.23; P = 0.006 as shown in Fig. 11. All-cause death, MACEs, TVR and TLR were not significantly different between ZES and SES/PES with RR: 1.02, 95% CI: 0.75–1.39; P = 0.90, RR: 1.11, 95% CI: 0.93–1.34; P = 0.25, RR: 1.06, 95% CI: 0.78–1.43; P = 0.73 and RR: 1.00, 95% CI: 0.67–1.50; P = 0.98 respectively as shown in Fig. 12 and represented in Table 6.

**Table 6:** Comparison of outcomes according to the follow-up periods.

Follow-up (years)	Total number of patients treated with ZES (n)	Total number of patients treated with SES, PES or EES (n)	Type of DES versus ZES	Analytic report (RR with 95% CI)
2 years	1222	1213	SES versus ZES	No significant difference was observed in ST
2 years	1383	1384	PES versus ZES	
2 years	2100	2098	EES versus ZES	—
3 years	150	154	SES versus ZES	—
4 years	1140	1152	EES versus ZES	—
4 years	1174	1236	SES versus ZES	Definite or probable ST significantly favored ZES [1.98 (1.22–3.23); p = 0.006
5 years	1469	1278	SES versus ZES	
5 years	921	915	PES versus ZES	

Abbreviations: ZES: zotarolimus eluting stents, SES: sirolimus eluting stents, PES: paclitaxel eluting stents, EES: everolimus eluting stents, DES: drug eluting stents, RR: risk ratios, CI: confidence intervals, ST: stent thrombosis.

### Long-term adverse outcomes which were observed with ZES versus EES

**Figure 13:**
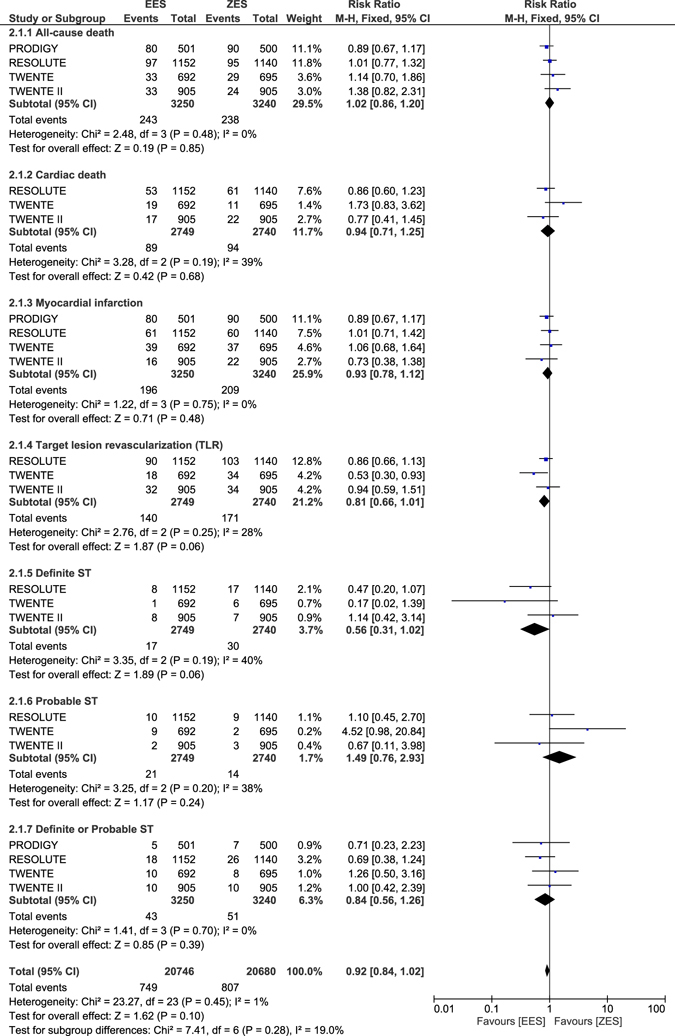
Long-term adverse outcomes which were observed with ZES versus EES (part 1).

**Figure 14:**
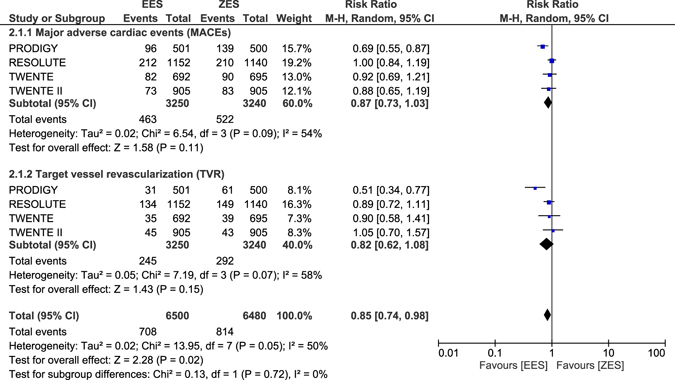
Long-term adverse outcomes which were observed with ZES versus EES (part 2).

When ZES were compared with EES during the long-term follow-up, no significant difference was observed in clinical outcomes such as all-cause death, cardiac death, MI, TLR, definite ST, probable ST, definite or probable ST, MACEs and TVR with RR: 1.02, 95% CI: 0.86–1.20; P = 0.85, RR: 0.94, 95% CI: 0.71–1.25; P = 0.68, RR: 0.93, 95% CI: 0.78–1.12; P = 0.48, RR: 0.81, 95% CI: 0.66–1.01; P = 0.06, RR: 0.56, 95% CI: 0.31–1.02; P = 0.06, RR: 1.49, 95% CI: 0.76–2.93; P = 0.24, RR: 0.84, 95% CI: 0.56–1.26; P = 0.39, RR: 0.87, 95% CI: 0.73–1.03; P = 0.11 and RR: 0.82, 95% CI: 0.62–1.08; P = 0.15 respectively as shown in Figs 13 and 14.

**Figure 15:**
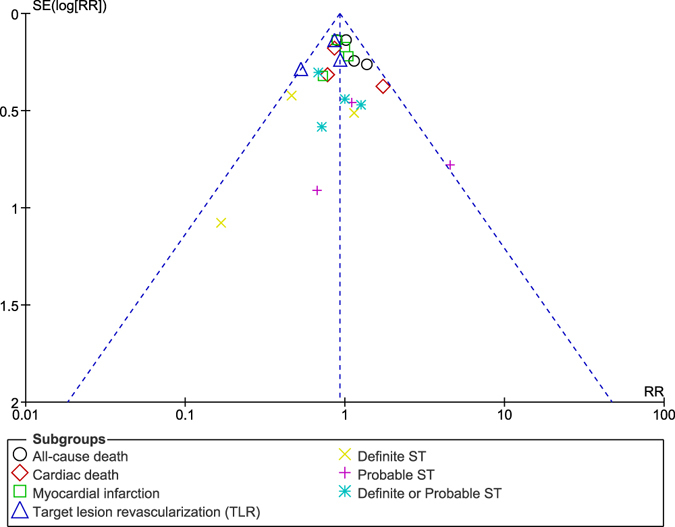
Funnel plot representing publication bias (**A**).

**Figure 16:**
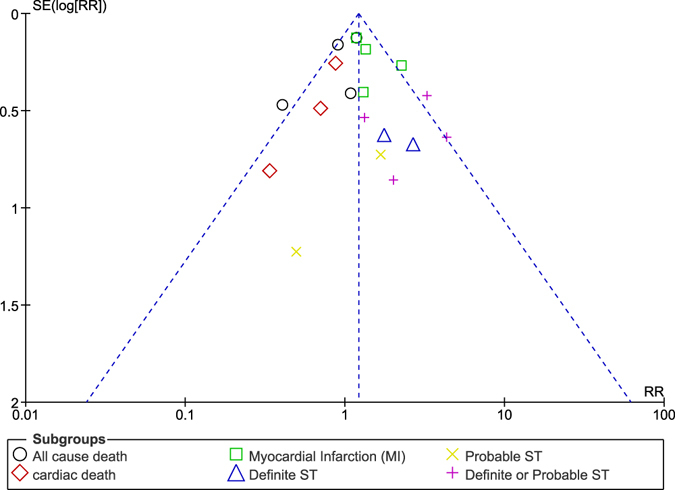
Funnel plot representing publication bias (**B**).

**Figure 17:**
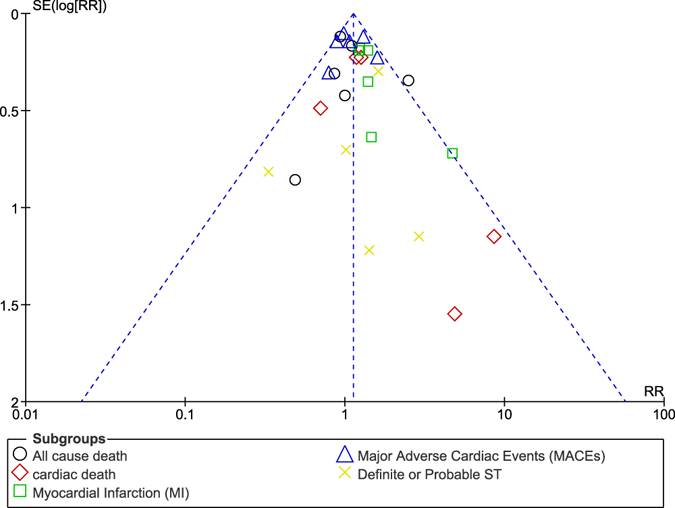
Funnel plot representing publication bias (**C**).

The funnel plots which were obtained showed a low evidence of publication bias across the trials that were involved when assessing the primary and secondary outcomes (Figs 15, 16 and 17).

## Discussion

Since many previously published studies comparing ZES with SES, PES and EES had a follow up period which was restricted to only one year, further studies with longer follow up periods were required to assess ST (a major shortcoming of DES) and other adverse clinical outcomes following PCI with these DES.

EES and ZES also did not show any significant difference in outcomes during the long term. However, this current analysis showed ZES to be associated with a significantly lower long-term definite or probable ST compared to SES or PES. Long-term definite ST was also significantly higher with SES and PES. This difference was more prominent when SES and PES were combined and analyzed. However, when these two types of DES were separately compared with ZES, a significantly higher risk of ST was mainly associated with PES. But, when other adverse clinical outcomes were compared, the risk of mortality, repeated revascularization and MACEs were similarly observed with these different types of DES.

One of our recent meta-analysis comparing ZES with EES at 1 year follow-up showed both DES to be associated with similar adverse clinical outcomes^27^. Even during a longer follow up period, no significant differences were observed with these two types of second-generation DES as shown in this current analysis.

Previously, Sethi *et al*. showed that ZES were non-superior to PES but they were inferior to SES in terms of angiographic outcomes and repeated revascularization^10^. However, even if their analysis consisted of 7 trials, only one trial had a longer follow up period of 3 years, whereas two trials had a follow up period of 2 years while the remaining trials had a follow up of only 18 months or one year. However, it should not be ignored that this current analysis which involved 12 trials, had a mean follow up period ranging from 2 years to 5 years, which might have been responsible for the difference in the results obtained.

Another meta-analysis comparing ZES with SES showed the latter to be superior to the former in reducing TLR and MACEs whereas TVR, ST and mortality were similarly observed^11^. However, the analysis only had a short-term follow-up period of 12 months in comparison to this current analysis which also included participants who were treated with PES for a longer time period.

Nevertheless, the PROTECT trial^12^ showed results which were partly similar to this analysis. PROTECT trial which compared ZES with SES for a long-term follow up period of 4 years, and involving a very large number of patients (more than 8000 patients), showed definite or probable ST to be significantly higher with SES (2.6%) compared to ZES (1.6%). A higher rate of very late (>1 year) ST was also seen to be associated with SES and this difference was observed especially from 3^rd^ to 4^th^ year onwards after PCI (1.8% at 3^rd^ year and 2.6% at 4^th^ year) with SES compared to (1.4% at 3^rd^ year and 1.6% at 4^th^ year) with ZES. This decreased ST associated with ZES was gradually achieved over years. However, even if the mortality rate and TLR were also minimal with ZES, this current analysis did not show any difference in these outcomes, except for a significantly lower risk of MI which was associated with ZES.

The ENDEAVOR IV trial including 722 patients who were treated with ZES and 718 patients who were treated with PES, also showed that at 5 years, very late ST were significantly lower with ZES when compared to PES (0.4% versus 1.8%)^17^. Significant improvement in late ST was observed with ZES. However, the authors suggested that this result should be considered hypothesis-generating, due to the limited statistical power of their research. But, it should be carefully noted that this current analysis further confirmed the results which were obtained in the ENDEAVOR trial, with a larger number of patients.

Furthermore, another very important observation was made with the SORT OUT III trial^20^. At one year, ZES were associated with a higher rate of definite ST (1.1% with ZES compared to 0.3% compared to SES). However, a completely different result was observed between 1 to 5 years follow up. A higher rate of ST was observed in the SES group compared to the ZES group during this longer follow up period (1.8% with SES and 0.1% with ZES) and the authors concluded that this superiority of SES compared to ZES at 1 year follow up was later lost after 5 years. These major observations are very important clinically and long-term follow up of ST defined by ARC must again be reviewed with ZES, SES, PES and EES.

### Novelty

Many previously published analyses had a follow up period restricted to one year and further research with longer follow up periods were required. This analysis compared the clinical outcomes which were observed with ZES versus SES, PES and EES during the long-term (2–5 years) showing a very strong plus point which at least responded and provided an answer to the limitations and recommendations of several previously published studies. In addition, the NNTs and the OIS were also calculated. According to the OIS, the minimum amount of information required to reach this reliable conclusion about definite or probable ST and several other adverse outcomes was sufficient.

### Limitations

A small number of participants which were included could be one limitation of this analysis. A moderate level of heterogeneity was observed when analyzing definite ST whereas a high level of heterogeneity was observed when analyzing MACEs and repeated revascularization which could have been due to selection and publication bias. In addition, SES and PES were combined and compared to ZES further contributing to the limitations. However, this issue was resolved when SES and PES were separately analyzed. Moreover, different long-term follow-up periods reported in this analysis (few trials had a follow up period of 2 years, 3 years, 4 years and 5 years respectively) could also have influenced the results. This issue was also addressed when all the trials having a follow-up period of 2 years and 5 years respectively, were separately analyzed. Nevertheless, outcomes at 3 years and 4 years respectively, could not be assessed because only one trial each had such follow-up periods, and there was no other trial for comparison. At last, even if funnel plots were used to represent publication bias in this analysis, due to a small study effect, the Harbord Test could probably better represent publication bias in this analysis.

## Conclusions

During this long-term follow-up period (2 to 5 years), ZES were associated with a significantly lower definite or probable ST compared to SES or PES. MI was also significantly lower with ZES. However, other adverse clinical outcomes were not significantly different between these two types of drug-eluting coronary stents. Even when ZES were compared to EES, no significant difference in adverse outcomes were noted during this longer follow-up period. Future research should be able to confirm this hypothesis.
